# Influence of Different Build Orientations and Heat Treatments on the Creep Properties of Inconel 718 Produced by PBF-LB

**DOI:** 10.3390/ma16114087

**Published:** 2023-05-31

**Authors:** Anke Kaletsch, Siyuan Qin, Christoph Broeckmann

**Affiliations:** 1 Institute for Materials Applications in Mechanical Engineering (IWM), RWTH Aachen University, Augustinerbach 4, 52062 Aachen, Germany; 2 Institute of Applied Powder Metallurgy and Ceramics at RWTH Aachen e.V. (IAPK), Augustinerbach 4, 52062 Aachen, Germany

**Keywords:** PBF-LB, Inconel 718, creep, HIP, post-processing, hot isostatic pressing

## Abstract

Inconel 718 is a nickel-based superalloy with excellent creep properties and good tensile and fatigue strength. In the field of additive manufacturing, it is a versatile and widely used alloy due to its good processability in the powder bed fusion with laser beam (PBF-LB) process. The microstructure and mechanical properties of the alloy produced by PBF-LB have already been studied in detail. However, there are fewer studies on the creep resistance of additively manufactured Inconel 718, especially when the focus is on the build direction dependence and post-treatment by hot isostatic pressing (HIP). Creep resistance is a crucial mechanical property for high-temperature applications. In this study, the creep behavior of additively manufactured Inconel 718 was investigated in different build orientations and after two different heat treatments. The two heat treatment conditions are, first, solution annealing at 980 °C followed by aging and, second, HIP with rapid cooling followed by aging. The creep tests were performed at 760 °C and at four different stress levels between 130 MPa and 250 MPa. A slight influence of the build direction on the creep properties was detected, but a more significant influence was shown for the different heat treatments. The specimens after HIP heat treatment show much better creep resistance than the specimens subjected to solution annealing at 980 °C with subsequent aging.

## 1. Introduction

Powder bed fusion with laser beam (PBF-LB) is a widely researched and utilized additive manufacturing process that uses a high-power laser to selectively melt metal powder layerwise, thereby producing components with the highest geometric freedom in a layer-by-layer structure [1,2,3]. PBF-LB produced samples typically present an anisotropic microstructure due to epitaxial grain growth in the build direction, resulting in anisotropic mechanical properties [2,4]. Aerospace engineering has a strong interest in beam-based additive manufacturing processes like PBF-LB or powder bed fusion with electron beam (PBF-EB), focusing on processing titanium alloys, titanium aluminides, and nickel-based alloys [5,6,7,8]. 

Inconel 718, a precipitation hardening and solid solution strengthened nickel-based superalloy, is commonly used in high-temperature applications, such as gas turbine disks and combustors, due to its excellent creep properties, good tensile and fatigue strength, and corrosion resistance [9,10]. The matrix of Inconel 718 consists of Ni-Fe-Cr austenite (γ), with very fine γ′-(Ni_3_(Al,Ti)) and γ″-(Ni_3_Nb) precipitates dispersed in the γ-matrix after heat treatment for strengthening. γ″ is metastable and has an identical chemical composition to the stable δ-phase. However, δ-phase cannot contribute to the strength of the matrix because, unlike the cubic γ″-phase, the orthorhombic δ-phase is incoherent with the γ-matrix. In addition, its needle-like shape might have a notch effect in the microstructure and, as a result, have a negative influence on the fatigue strength [11]. Additionally, when δ-phase forms, it consumes Nb from the matrix, leading to a loss of γ″-precipitates and strength [12]. However, if δ-phase precipitates at the grain boundaries, which is mostly the case, it suppresses grain growth [13] and affects the grain boundary creep fracture [14]. The grain growth inhibiting effect of the δ-phase is particularly desirable when forging Inconel 718.

Although plenty of publications exist on the PBF-LB manufacturing of nickel-based alloys, the creep strength of additively manufactured nickel superalloys has not yet been extensively researched. In this context, most publications exist on the creep of Inconel 718 [15,16,17,18,19]. The influence of the typical PBF-LB microstructure, mentioned above, on the creep behavior has already been described [20]. Moreover, some investigations have been made on the influence of heat treatment. Using the AMS 5662 standard heat treatment for additively manufactured Inconel 718, which includes solution annealing at 980 °C and double aging at 720 °C and 620 °C, results in a lower creep strength than a forged reference due to the high content of the Laves- and δ-phase [21]. The Laves phase is found in the as-built condition due to Nb segregation in the dendrite interstices. Solution annealing at 980 °C is not sufficient to completely dissolve the Laves phase and homogeneously dissolve and distribute Nb in the matrix. Therefore, a large number of acicular δ-phase forms within the crystallites in regions with increased Nb content. By consuming Nb, the δ-phase reduces the formation of the γ″-phase, which is needed for superior creep properties. The very coarse δ-phase at the grain boundaries can also have an unfavorable effect on the creep properties [22]. Therefore, increasing the solution temperature in the heat treatment can improve the creep properties [23,24].

Hot isostatic pressing (HIP) is a process in powder metallurgy that consolidates metal powder filled in a metallic capsule into solid materials by simultaneously applying high pressure and high temperature [25]. This technology can also be used to consolidate materials with pores or voids to full density. For example, cast turbine blades for safety-relevant aerospace applications are post-densified with HIP as standard to guarantee high mechanical properties and high reliability. The HIP process is also suitable for the post-densification of additively manufactured materials and, thus, optimizing their mechanical properties. In particular, fatigue strength can be significantly increased via HIP post-treatment [26,27,28,29,30,31], due to minimizing the fatigue critical defects like the lack of fusion (LOF), microcracks, and porosity. HIP can also have a positive effect on the creep properties of PBF-LB manufactured materials. Kuo et al. [23] and Qin et al. [32] showed that HIP can improve the creep life of PBF-LB manufactured Inconel 718. This can be attributed, on the one hand, to the fact that the solution annealing temperature in HIP treatment is higher than in conventional heat treatment. High HIP temperatures, for nickel-based superalloys between 1100 °C and 1280 °C [25], are generally required to achieve full densification of the material at the applied pressure. This leads to a different solution state of the alloying elements and possibly grain growth. On the other hand, the defects typical for PBF-LB, such as LOF, cracks, and pores, lower the creep rupture strain. Thus, densification of these defects can improve the creep properties.

Nonetheless, HIP usually also leads to a homogenization of the microstructure, which is maybe not advantageous in the case of creep properties. The elongated grains resulting from epitaxial grain growth could have a positive influence on the creep behavior in the build direction. Rickenbacher et al. [33] have shown in creep tests on IN738LC that the columnar grain structure resulting from epitaxial grain growth in the PBF-LB process leads to better creep properties for specimens built vertically compared to those built horizontally. Homogenization of the columnar grain structure during HIP could, thus, also have an effect on the orientation dependency of the creep properties.

Therefore, the aim of the present study was to investigate the orientation dependency of the creep properties of Inconel 718 produced via PBF-LB. Furthermore, it considered whether a HIP post-treatment influences the orientation dependency of the creep properties. For this purpose, two different build orientations were investigated to evaluate the influence of the anisotropic PBF-LB microstructure. Then, HIP heat treatment with integrated quenching and subsequent aging was performed on one half of the samples. 

As a reference, the other half of the specimens were subjected to a conventional standard heat treatment (AMS 5662). In addition to the microstructure, the rupture characteristics of the fractured specimens were evaluated after the creep tests and correlated with the obtained creep properties.

## 2. Materials and Methods

### 2.1. Material and PBF-LB Process

**Figure 1:**
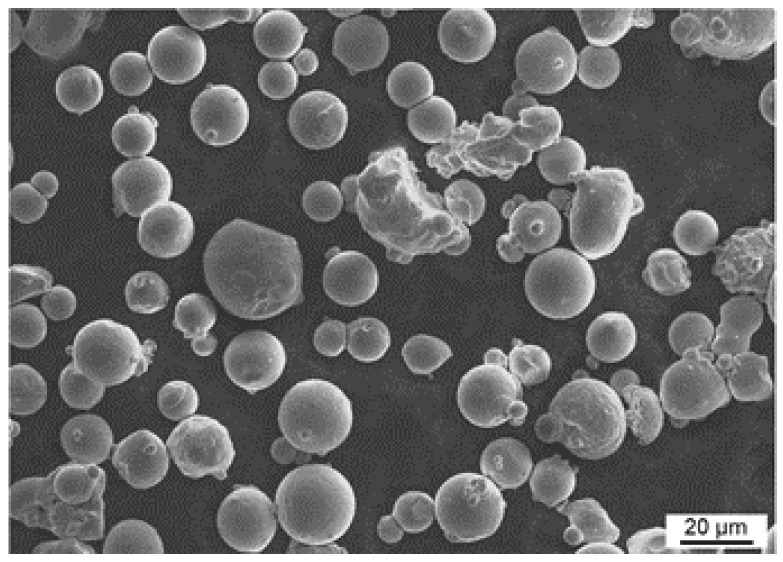
The morphology of the IN718 powder used in this study.

Argon-atomized Inconel 718 (IN718, Alloy 718, EN NiCr19NbMo, 2.4668) powder was used for this study and supplied by Carpenter Technology Corporation. The chemical composition of the powder was in accordance with the ASTM B637-18 specification [34], as shown in Table 1. The powder particles had a spherical shape (see Figure 1) and a particle size in the range of 15 to 45 μm. 

**Figure 2:**
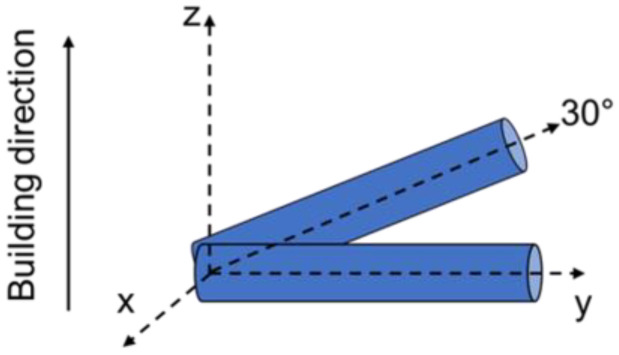
Schematic representation of the different build orientations. The cylindrical specimens were built either horizontally or at an angle of 30°.

The creep specimens were produced under an argon atmosphere with a ReaLizer SLM 100 machine (ReaLizer GmbH, Borchen, Germany) utilizing a ytterbium fiber laser. The parameters for the PBF-LB fabrication of IN718 were taken from a previous study [35]: a laser power of 160 W, a laser scanning speed of 1000 mm/s, and a hatch distance of 100 µm were used. With these parameters, cylindrical specimens with a diameter of 11.5 mm were built up in two different orientations, either horizontally or at an angle of 30° with respect to the build direction, as depicted in Figure 2. For the analysis of the microstructure before creep testing, additional cubic specimens with an edge length of 10 mm were built.

### 2.2. Heat Treatment

**Figure 3:**
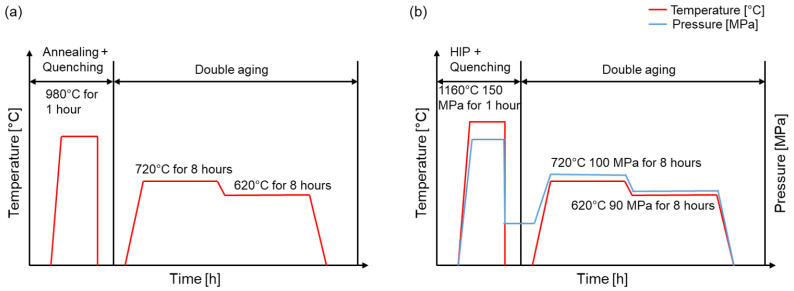
Graphical representation of the temperature profiles for the two heat treatments used in this study: (**a**) heat treatment for IN718 according to AMS 5662; (**b**) HIP heat treatment with integrated quenching and aging under pressure.

Different heat treatment conditions were investigated for the specimens built in different orientations. In one variation, a usual standard heat treatment for IN718 was performed, which means that half of all the specimens were heat treated according to the AMS 5662 standard. This heat treatment included solution annealing at 980 °C for 1 h, followed by quenching in water. Subsequent double aging was performed at 720 °C for 8 h, followed by furnace cooling at 50 K/h and 620 °C for 8 h, followed by air cooling, as shown in Figure 3a. 

The second half of the specimens were heat treated under pressure in the HIP with a Uniform Rapid Cooling (URC©) furnace at Quintus Technologies Application Centre (Västerås, Sweden). Typical HIP parameters for IN718 were utilized, with a HIP temperature set to 1160 °C at 150 MPa for 4 h. This was followed by gas quenching and a double aging treatment at 710 °C and 100 MPa for 8 h, and 610 °C and 90 MPa for 8 h, as shown in Figure 3b.

### 2.3. Microstructure Characterization

To study the microstructure before the creep testing, the cubic specimens were mechanically ground, polished and then etched with Kalling’s 2 reagents for 5 min. Microstructural analyses were performed using a light optical microscope (LOM) Zeiss ‘Axio Imager M2m’ (Carl Zeiss AG, Oberkochen, Germany) and Leica DM4000 (Leica Microsystems GmbH, Wetzlar, Germany) and a scanning electron microscope (SEM) Helios Nanolab G3 CX (FEI, Hillsboro, OR, USA). The porosity of the specimens was determined using image analysis on the unetched cross-sections.

### 2.4. Creep Testing and Fracture Analysis

**Figure 4:**
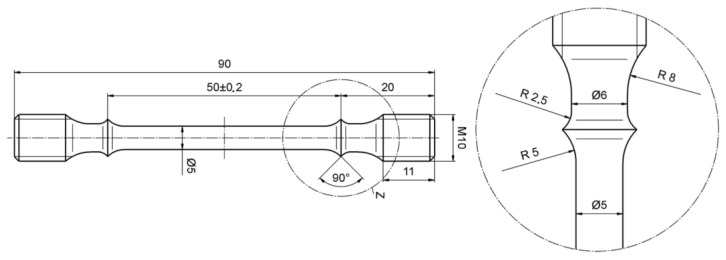
Specimen geometry for the creep tests.

The creep tests were performed at four different stress levels of 130 MPa, 170 MPa, 210 MPa, and 250 MPa, and a temperature of 760 °C. The specimens were machined to the geometry required for the creep test, with a gauge length of 50 mm, and a diameter of 5 mm, as shown in Figure 4. A lever-arm creep testing machine was used for the creep test, leading to loading with a constant axial force during the test, according to ASTM E139-2011 [36]. After testing, the fracture surfaces were examined, and the microstructures were analyzed using SEM. Due to the extended duration of creep tests, substantial noise is present in the raw data. MATLAB was employed to filter the noise and process the data effectively. 

## 3. Results and Discussion

### 3.1. Microstructure

**Figure 5:**
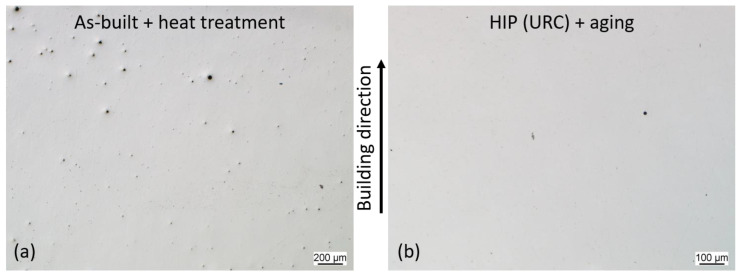
Cross-sections of the specimen: (**a**) as-built; (**b**) after HIP.

The polished cross-sections of the unetched specimens revealed spherical, PBF-LB process-induced pores in the as-built condition, as depicted in Figure 5a. The as-built porosity was determined via image analysis to be 0.083%. After HIP treatment, almost all the pores were densified, resulting in a porosity of 0.04%. A few small pores can still be seen after the HIP process, see Figure 5b.

**Figure 6:**
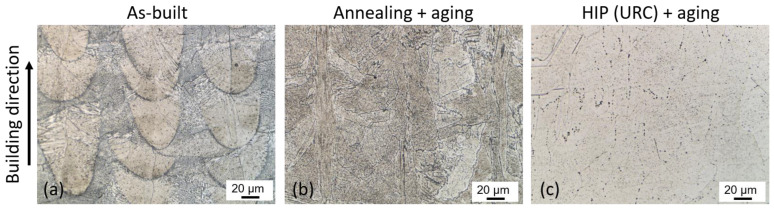
LOM images of the etched specimens: (**a**) as built; (**b**) after annealing at 980 °C and aging; (**c**) after HIP with integrated quenching and aging.

After chemical etching, the microstructure can be revealed in more detail. Figure 6a illustrates the melt pools and layer formation resulting from an alternating (x-y) layer-by-layer melt scan. The average layer thickness is approximately 50 µm, while the width of the melt pools varies between 70 and 120 µm. The grains exhibit an elongated shape in the build-up direction, resulting from the PBF-LB typical epitaxial grain growth [14]. The dendritic structures observed within the grains are a consequence of rapid cooling during the PBF-LB process. Segregations of the alloying elements can be seen in dark gray at the lower edges of the melt pools. 

After the standard heat treatment with solution annealing and aging, the melt pools are dissolved, but the elongated grains remain (Figure 6b). In contrast, after HIP and aging, the microstructure resulting from the PBF-LB process is dissolved and homogenized, as shown in Figure 6c. The black dots that can be seen in Figure 6c are carbides that were not dissolved in the matrix during the HIP process. These carbides can be observed at the previous grain boundaries of the PBF-LB microstructure, where they formed during the PBF-LB process.

**Figure 7:**
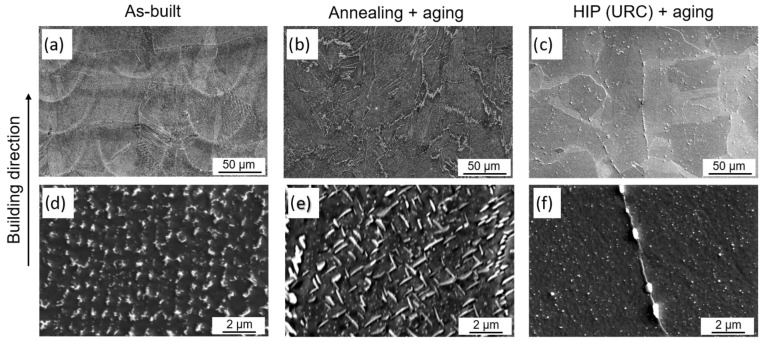
SEM images of the microstructure in different conditions: (**a**,**d**) as-built; (**b**,**e**) after standard heat treatment; (**c**,**f**) after HIP with integrated quenching and aging.

The microstructure of the specimens was further analyzed in detail using SEM. Figure 7 presents the different microstructural conditions: in the as-built condition (Figure 7a,d), a fine solidification structure can be observed inside and around the melt pools, while the structure outside the melt pools is coarser. The interdendritic phase in the PBF-LB samples, visible as white spots in Figure 7d, was assumed to be a Laves phase because this is consistent with descriptions from the literature [37,38]. The Nb-rich Laves phase is formed during the rapid solidification of the PBF-LB process, due to Nb segregation in the interdendritic regions. The standard AMS 5662 heat treatment cannot dissolve the Laves phase due to the too low solution annealing temperature of 980 °C and leads to the formation of intergranular acicular δ-phase (Ni_3_Nb) from the Nb-rich regions of the Laves phase (Figure 7b,e). However, the HIP process can completely dissolve both the Laves- and the δ-phase due to the high solution heat treatment temperature of 1160 °C, resulting in a homogeneous distribution of Nb in the matrix. In addition, the rapid cooling during HIP prevents further δ-phase precipitation. As depicted in Figure 7c,f, the microstructure after HIP and aging does not display any δ-phase. Instead, small white spheres are observable at the grain boundaries, representing the carbides previously mentioned. Additionally, even smaller white dots can be observed in the matrix, which could be carbides or potentially γ′ precipitates, as presented in Figure 7f. The γ″ precipitates, on the other hand, are too small to be seen with the SEM used in this study. However, it can be assumed that due to the dissolution of the Laves- and δ-phase and, thus, a high Nb content in the matrix, a high proportion of γ″ phase is precipitated during the heat treatment used in the HIP process. However, even though the material was recrystallized during HIP, the carbides still map the grain morphology of the PBF-LB microstructure, indicating that they did not completely dissolve during the HIP.

These results on the microstructure are in very good agreement with the results from a previous study [35], on additively manufactured Inconel 718 using the same PBF-LB and heat treatment parameters.

### 3.2. Tensile Creep Results

**Figure 8:**
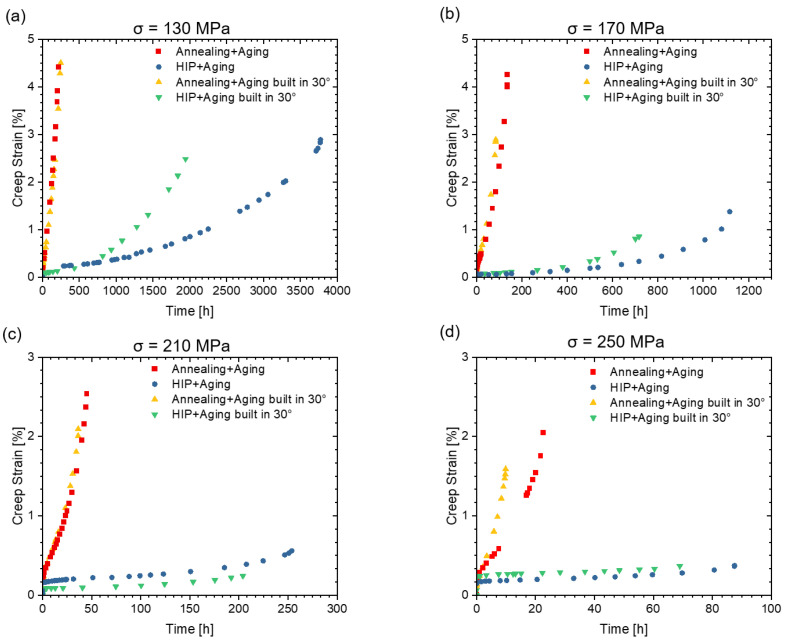
Creep curves of the specimens in different heat treatment conditions. The creep tests were conducted at 760 °C with four different stresses, specifically (**a**) 130 MPa, (**b**) 170 MPa, (**c**) 210 MPa, and (**d**) 250 MPa.

**Figure 9:**
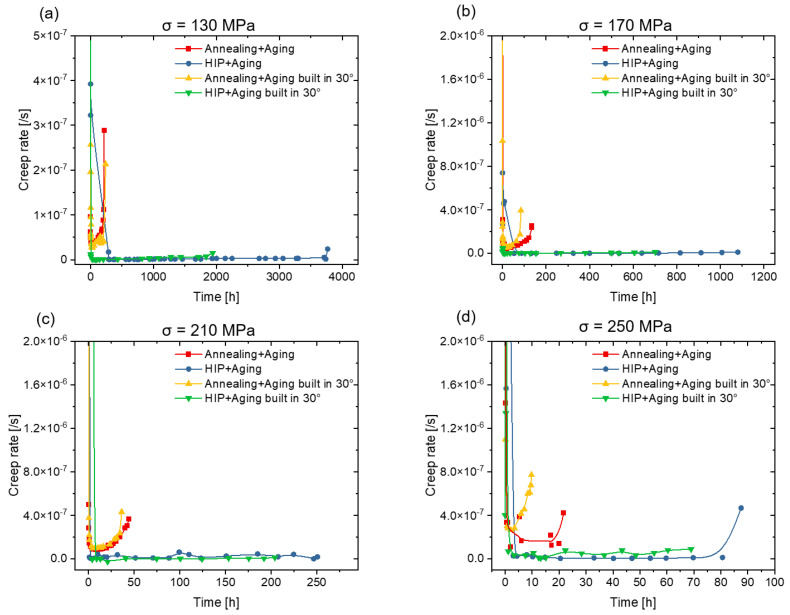
Creep rates of the specimens in different heat treatment conditions. The creep tests were conducted at 760 °C with four different stresses, specifically (**a**) 130 MPa, (**b**) 170 MPa, (**c**) 210 MPa, and (**d**) 250 MPa.

The creep performance for the different conditions was investigated at a constant temperature of 760 °C with four different stress levels from 130 MPa to 250 MPa. The creep curves in Figure 8 and the creep rates in Figure 9 exhibit a typical creep behavior for metallic material: after an initially unsteady plastic deformation, where the material starts to flow, a steady-state creep region with a constant deformation rate is reached and, finally, the deformation accelerate until failure.

Figure 8a and Figure 9a show the creep curves at a stress of 130 MPa. The specimens after HIP present a lower strain rate and a longer time to rupture than the specimen from the group with conventional heat treatment. The influence of build direction in the group with conventional heat treatment is not significant at the lower stress levels, while the HIP post-treated specimens built horizontally creep slower than those built at an angle of 30°. As the creep stress increases, the difference in creep rate, as indicated in Figure 9, between the two groups becomes more pronounced: the HIP post-treated specimens creep more slowly and last a longer time before fracture, suggesting a better creep resistance. At the stress level of 250 MPa (Figure 8d), the influence of build direction in the conventional heat treatment group becomes apparent, and the horizontally built specimens exhibit better creep resistance, consistent with the results of the HIP post-treated group.

**Figure 10:**
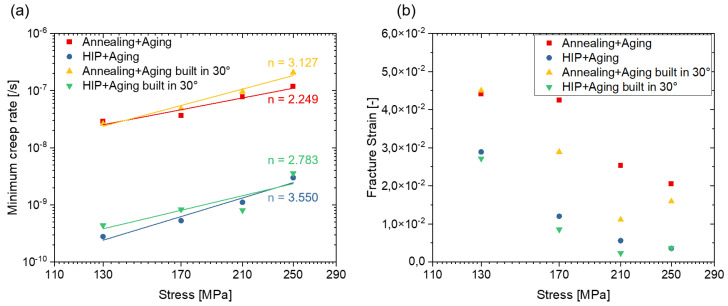
The dependence of the applied stress on the minimum creep rate (**a**) and fracture strain (**b**).

The minimum creep rate of the specimens is shown in Figure 10a. In general, the HIP-treated specimens, which contain fewer δ-phase and, thus, expectedly a larger amount of fine γ″ precipitates in the matrix, exhibit lower minimum creep rates at the applied stresses compared to the conventionally heat-treated specimens. The Norton creep law can be used to describe the minimum creep rate:

$$\dot{\varepsilon }=A\cdot \sigma^{n}$$

where $\dot{\varepsilon }$ is the minimum creep rate, *A* is a material constant, *σ* is the tensile stress, and *n* is the creep stress exponent. The stress exponent provides information about the mechanism that dominates creep deformation. 

The stress exponents of the specimens in this study range from 2.245 to 3.55, indicating dislocation creep as the dominant mechanism. The higher proportions of the uniformly distributed fine γ″ precipitates, expected in the HIP specimens, limit the mobility of the dislocations and may, therefore, be one reason for the higher creep strength in the HIP heat treatment condition.

Figure 10b presents the fracture strain at different stresses. Higher stress levels result in lower elongation at fracture due to lower plastic deformation. This can be explained by the fact that at low stress levels, void nucleation happens significantly later leading to increased rupture time and allowing more time for creep deformation. Therefore, specimens tested under low stresses present higher fracture strain. Furthermore, the fracture strain is higher in the conventionally heat-treated group than in the HIP heat-treated group, which will be further discussed in the next chapter.

**Figure 11:**
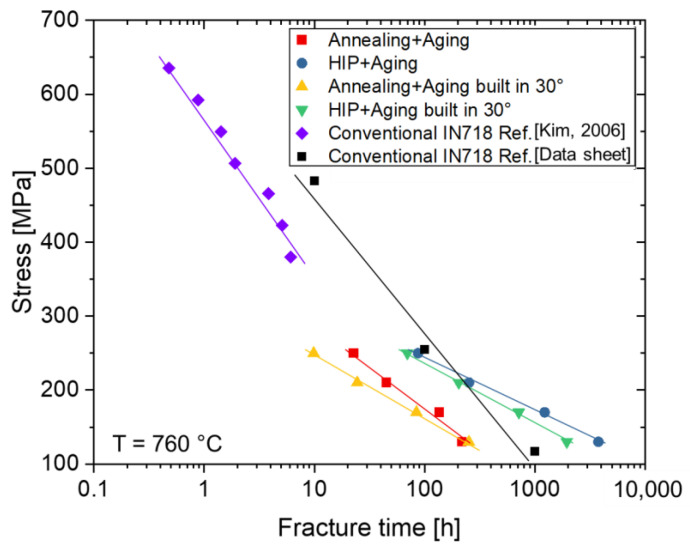
The fracture time in various conditions in this study compared with conventionally produced IN718 in references [39,40].

The fracture time for the specimens in various conditions in this study is compared with conventionally produced IN718 references [39,40], as shown in Figure 11. The HIP post-treated group shows a higher creep resistance, in particular a longer fracture time than the group with conventional heat treatment. In addition, the creep resistance of the specimens fabricated under 30° is worse than that of the horizontally fabricated specimens. Compared to conventionally fabricated IN718 [39,40], the HIP group exhibits even better creep resistance. The reasons will be discussed in more depth in the further sections, when analyzing the fracture characteristics.

A few studies already describe the influence of the build direction on the creep strength in the horizontal and vertical directions [20,41,42,43]. These studies have consistently shown that specimens built vertically exhibit better creep resistance compared to horizontally built specimens. This is attributed to the <001> texture and elongated grains along the build direction, resulting also in a vertically aligned grain boundary δ-phase. When the specimens are loaded horizontally, the grain boundary δ-phase can act as sites for the rapid formation of creep voids, leading to accelerated rupture. Hilal et al. [44] also found in creep tests on the Ni-based superalloy CM247LC produced via PBF-LB that specimens built at an angle of 30° exhibit poorer creep properties than specimens built vertically. As a reason for the better creep properties in the vertically built samples, they also attribute the columnar grain shape resulting from epitaxial grain growth. However, these theories do not explain the lower creep resistance in the 30° build direction compared to horizontally built specimens. One possible explanation is that the largest shear stresses and, thus, the strongest grain boundary slip occurs in general at 45° grain boundaries in the direction of the applied stress. If grain boundary sliding is assumed to be one of the creep mechanisms present, then creep damage will occur at those grain boundaries with the most creep and stress superelevation. Since epitaxial grain growth occurs in the PBF-LB process, the specimens built horizontally have most of the grain boundaries at an angle of 90° in the direction of loading. In contrast, within the specimens built at an angle of 30°, most of the grain boundaries have an orientation of 60° to the loading direction. This may result in higher shear stresses and more grain boundary slip, for these specimens.

### 3.3. Failure Analysis

**Figure 12:**
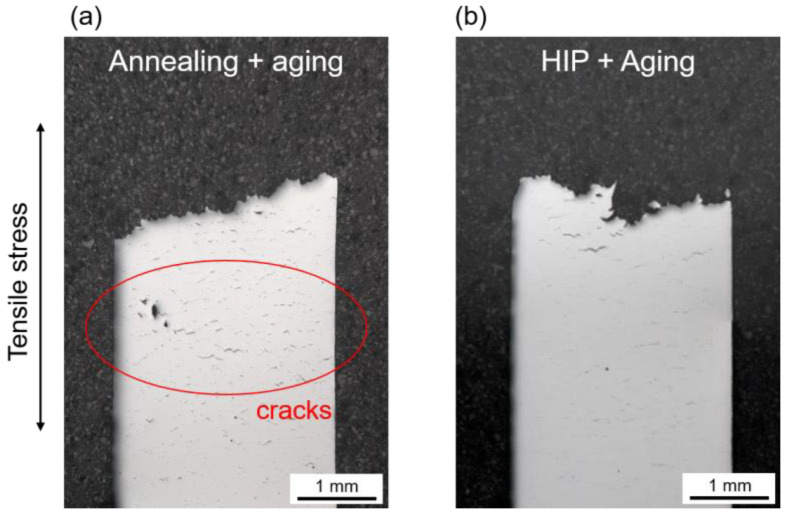
Representative cross-sections of the creep specimens after fracture. (**a**) Specimen built horizontally with standard heat treatment, tested at 250 MPa; (**b**) specimen built horizontally with HIP integrated heat treatment, tested at 250 MPa.

Figure 12 shows exemplary post-fracture cross-sections for two creep specimens tested at a stress of 250 MPa.

Both specimens were built in the horizontal orientation. Figure 12a shows a conventionally heat-treated specimen, while Figure 12b shows a specimen with HIP heat treatment. First, it should be mentioned that all samples show creep fractures without significant necking. Microcracks developed throughout the whole volume until void coalescence finally determined the main fracture. 

**Figure 13:**
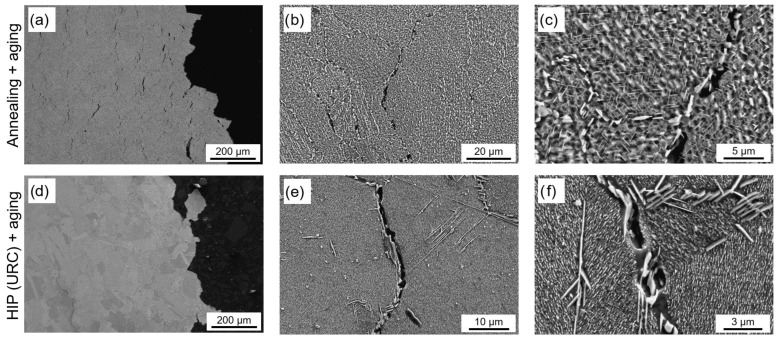
SEM images of the microstructure from the specimens tested at 250 MPa after creep testing. (**a**–**c**) Specimen built horizontally with standard heat treatment; (**d**–**f**) specimen built horizontally with HIP heat treatment.

Compared to the HIP heat-treated specimen, the conventionally heat-treated specimen exhibited a higher number of cracks and pores near the fracture surface. These cracks and pores presumably contributed to the final elongation of the specimens, resulting in a higher fracture strain in the conventional heat-treated group (Figure 10b). On the other hand, the lower number of cracks and pores in the HIP heat-treated group indicated a better creep resistance. The formation of cracks and pores can be attributed to the accumulation of plastic strains and the initiation and growth of microvoids, which are strongly influenced by the microstructure of the material. The microstructure of the specimens after creep testing is shown in Figure 13.

Due to the high temperature, the microstructure changed during the creep tests, resulting in the degradation of the material. Creep damage can generally be divided into several types, the two most important being: transgranular creep damage and intergranular creep damage. Which mechanism is dominant depends, on the one hand, on the temperature and the applied stress and, the other hand, on the creep properties of the material. In transgranular creep fracture, voids and cracks form within the matrix during the creep process due to the high temperature and stresses. Figure 13d shows that the HIP heat-treated sample contains nearly no cracks and voids within the grains compared to the conventionally heat-treated sample, but only along the grain boundaries or phase boundaries, respectively, and, thus, shows no transgranular creep damage. 

**Figure 14:**
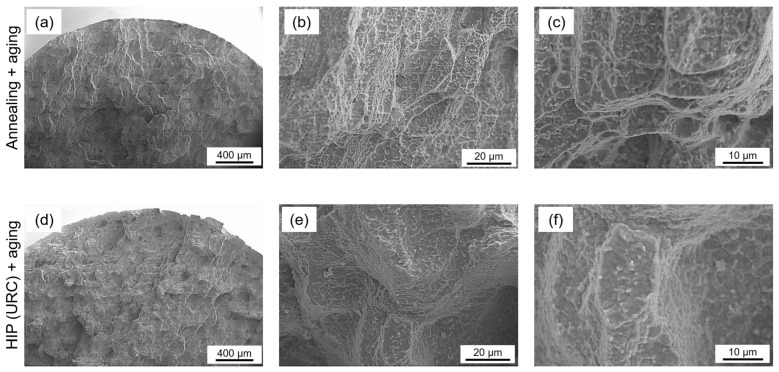
SEM images of the morphology of the fracture surfaces at different magnifications from specimens tested at 250 MPa. (**a**–**c**) Specimen built horizontally with standard heat treatment; (**d**–**f**) specimen built horizontally with HIP heat treatment.

Another type of failure is intergranular creep damage. In intergranular fracture, creep damage occurs at grain boundaries and triple points. In this case, the damage is often initiated at the crack nuclei, which during further development leads to microvoids and cavities, that later become cracks. Figure 13e shows that in HIP heat-treated specimens cracks are clearly visible at the grain boundaries (near δ-precipitates). In these specimens, long acicular δ-precipitates appear along the grain boundaries, which can act as crack nuclei and lead to intergranular fracture (Figure 14f). In contrast, in the conventionally heat-treated specimens, failure is evident not only at the grain boundaries, but especially within the grains leading to a certain amount of transgranular fracture. 

In all specimens, a coarsening of the γ″ precipitates can be observed, as shown in Figure 13c,f, which generally reduces the strengthening effect of the γ″ precipitates and leads to a decrease in the creep strength of the material.

The morphology of the specimens’ fracture surfaces after the different heat treatment conditions provides information about the deformation and failure mechanisms, as shown in Figure 14. The grain morphology observable in both fractures (Figure 14a,d) indicates the presence of intergranular fracture. The small dimples and pitting fracture surface of the conventionally heat-treated specimen in Figure 14b further indicate proportions of microscopic ductile fracture, while the fracture surface of the HIP post-treated specimen in Figure 14e is relatively smooth, indicating a lower degree of ductile deformation and a higher degree of intergranular fracture. Furthermore, coarsened precipitations can be observed on the fracture surface of both specimens (Figure 14c,f), indicating microstructure degradation.

## 4. Summary and Conclusions

In the present study, creep tests were performed on IN718 specimens built with PBF-LB in different orientations and subjected to two different heat treatments. The different build orientations were horizontal (0° to the build plate) and 30° inclined to the build plate. The heat treatment conditions were the conventional heat treatment according to AMS 5662 and the HIP heat treatment with integrated quenching and aging.

The study provides a description of the microstructural evolution in PBF-LB fabricated IN718. In the as-built initial condition, a large fraction of the Laves phase is visible, which is formed by Nb-segregations during rapid cooling during the PBF-LB process. The solution annealing temperature of 980 °C in the standard heat treatment was not sufficient to dissolve the Laves phase and homogenize the microstructure, resulting in a large amount of δ-phase within the matrix. In contrast, during the HIP treatment at 1160 °C, the matrix could be homogenized and both the Laves- and the δ-phase were dissolved. After HIP treatment with integrated quenching and aging, only the γ′ precipitates and carbides were observed. The γ″ precipitates present could not be resolved with the SEM used in this study, but it can be assumed that a high amount of γ″ precipitates is present after the HIP heat treatment.

The PBF-LB specimens exhibit lower minimum creep rates and longer life after HIP heat treatment than after conventional heat treatment, indicating better creep resistance. Microstructural fracture analysis shows that the specimens after HIP heat treatment exhibit mainly intergranular creep damage and little damage inside the grains. In contrast, the specimens with the conventional heat treatment exhibit a higher percentage of transgranular fracture, indicating that the creep damage was initiated also in the grain interior. Sites for crack initiation can be voids, such as pores, but also larger precipitates that are not coherent with the matrix, such as δ-phase. Both are present within the matrix of the specimens with conventional heat treatment. The samples after HIP heat treatment show a strongly reduced porosity and, also, no initial amount of δ-phase inside the grains. 

In addition, it can be assumed that the specimens after HIP heat treatment have a much larger fraction of creep-strengthening γ″ precipitates. The reason for this assumption is that due to the dissolution of the Laves- and δ-phase at the higher solution annealing temperatures in HIP, more Nb is available to form the γ″ precipitates in the matrix. Thus, after HIP heat treatment, the specimens exhibit a more creep-resistant matrix and, at the same time, fewer defects in the microstructure, which can act as crack initiation points. That leads to superior creep properties in the samples with HIP treatment. 

The specimens built horizontally present, for both heat treatment conditions, a better creep resistance than the specimens built at 30°. The reason for this is seen in the higher shear stresses at the grain boundaries for the 30° built samples. Due to the PBF-LB typical epitaxial grain growth in build direction, the orientation of the grain boundaries is less favorable to the load direction for a build direction of 30° than in a horizontal direction. The higher shear stresses at the grain boundaries lead to faster creep damage in this area.

Nevertheless, based on the results of the present study, it can be concluded that the influence of the PBF-LB build direction on the creep behavior is much smaller than the influence of the different heat treatments.

## Figures and Tables

**Table 1:** Chemical composition of the IN718 powder in comparison to the standard range of chemical composition according to ASTM B637-18 [34].

	C	Mn	Si	Cr	Ni	Mo	Nb	Ti	Al	Cu	Fe
Investigated Material	0.04	0.01	0.02	18.73	54.24	2.95	4.81	0.97	0.46	0.01	Bal.
ASTM Standard	<0.08	<0.35	<0.35	17–21	50–55	2.8–3.3	4.75–5.5	0.65–1.15	0.2–0.8	<0.3	Bal.

## Data Availability

The data presented in this study are available on request from the corresponding author.
